# The Domestic Management of the Sick Room, Necessary, in Aid of Medical Treatment, for the Cure of Diseases

**Published:** 1845-12

**Authors:** 


### BIBLIOGRAPHICAL NOTICES.

*The Domestic Management of the Sick Room, necessary, in aid of Medical treatment, for the cure of diseases.* By Anthony Todd Thompson, M. D., F. L. S., &c.—First American, from second London edition,—Revised, with additions by R. E. Griffith, M.D., &c. Philadelphia: Lea & Blanchard; pp. 353, 12 mo.—(From the publishers.)

As its title imports this work is intended for the instruction of the nurse, rather than the physician, though the latter may perhaps also receive from it much useful practical information, It is not, like various other works on domestic medicine, intended to *take the place* of the physician, but merely to supply that necessary information in the cure of the sick, in regard to comfort, administration of prescribed medicines, preparation of external applications, cookery for the convalescent, &c. &c., the want of which, in the attendants, is a source of such frequent annoyance to the practitioner, and of such fatal consequences to the patient. The work defines how far the nurse may go, and when the physician must be sent for, also a matter of importance. We give our approbation to the work, and express it as our opinion that every practitioner is personally interested in its general circulation among his employers. (For sale by Brautigam & Keen, Chicago.) ED.

